# Sketches in Bedlam; or Characteristic Traits of Insanity

**Published:** 1823-12-01

**Authors:** 


*Sketches in Bedlam; or, Characteristic Traits of Insanity, as displayed in the Cases of* 140 *Patients of both Sexes, now, or recently confined in New Bethlem, including Margaret Nicholson, James Hatfield, &c. &c. To which are added, a Succinct History of the Establishment, &c.* By a Constant Observer. Octavo, pp. 312. July, 1823.

"See how the noble mind is here o'erthrown."

It is with the mind as with the body, there is scarcely an individual, at least in civilized life, who is in possession of perfect *sanity*, whether mental or corporeal. Every human being has his peculiarities or whims. When these become rather prominent they are termed eccentricities—and these again rising a few grades, assume the formidable appellation of insanity. In this last state the mind exhibits as great a variety of symptoms as the body under disease. One man is playful, harmless, and, we doubt not, superlatively happy in madness—fancying himself a prince or other great personage, and enjoying, in imagination, all the honours, without any of the anxieties, of exalted rank. Another is morose or taciturn, busied with schemes of various kinds which will never be put in execution. A third is furious, and endangers the lives of his neighbours. All these things, however, pass before our eyes every day in common life—but being only in a certain degree of intensity, they are not viewed as aberrations from soundness of mind. In such a place as Bedlam we see exquisite specimens of the human intellect in its widest divergencies from the common standard; the same as in an hospital, we see a congregation of the most remarkable diseases of the body. A faithful sketch of the inmates of one of these infirmaries for the mind, cannot be uninteresting to society at large (however impolitic the publication of it) nor to the medical practitioner, whose duty and interest it is to study equally the phenomena of health and disease, in all parts and functions of the human fabric.

Old Bethlem, in Moor Fields, was built on the plan of the Royal Palace of the Tuilleries at Paris, which imitation gave such offence to the *grand monarche*, Louis XIV. that he ordered the plan of St. James's Palace to be taken for an office that shall be nameless! The present hospital is a noble pile of building erected on the site of the once celebrated DOG and DUCK TAVERN—a curious revolution, from scenes of voluntary madness excited by wine, to the straight-waistcoat and solitary cell of the present colony of maniacs! It is capable of containing 200 patients of both sexes, besides two wings for criminals, supported by Government, and calculated to accommodate sixty more. The asylum is supported partly by the Bridewell estates, and partly by voluntary contributions. The building and grounds occupy an area of about twelve acres. Physicians, Sir Geo. Tuthill, and Dr. Thomas Munro—Surgeon, W. Lawrence, Esq.—Apothecary and Superintendent, Dr. Ed. Wright. The patients rise every morning, in summer, at six, and in winter at seven o'clock. They breakfast at eight and half-past eight—dine at one—sup at six, and retire to bed at eight, when they are locked up.

In respect to treatment (moral) the grand principle is mildness, as being generally acknowledged the most fitting for the maniac. The breakfast throughout the year is wholesome gruel mixed with milk, and two ounces of bread. The dinner varies every day, and seems to average about half a pound of meat every other day, with good vegetable and farinaceous substitutes and auxiliaries in the intervals and on meat days. Excellent table-beer is allowed without any stint as to quantity. This, we think, is rather too liberal—for we have known many people get half-intoxicated or muddle-pated daily by the use or rather abuse of this beverage. The clothing, bedding, and indeed all the other regulations, appear to be excellent in their kind. It is strange that, notwithstanding the excellence of this establishment, and the supposed increase of insanity, there are generally a considerable number of vacancies—"although the only qualifications for admission here are insanity and poverty." The disqualifications are 1st. The possession of property sufficient for their support in a private asylum—or affluent relations. 2d. More than 12 months duration of the disease. 3d. The having been discharged uncured from some other asylum. 4th. Pregnancy. 5th. Idiotcy, palsy, or epilepsy. 6th. Syphilis or itch. 7th. Such debility from age as to require a nurse, or to threaten speedy dissolution.

A certificate from the minister, churchwardens, overseers, and a friend or relation, stating that the foregoing regulations have been read, and that the patient is not in any of the states or conditions therein specified, is required, together with a medical certificate from a physician, surgeon, or apothecary.

We now come to offer a few sketches of the inmates of this intellectual lazar-house to our readers, selecting those which are most interesting, and using all the brevity and terseness of language in our power.

1. Pat. Walsh. This ferocious maniac has been deranged altogether about 12 years, and has uniformly evinced a character of desperation, vengeance, and sanguinary cruelty, scarcely conceivable even in madness! He is more like a tiger than a human being. It is not improbable that a disposition naturally fierce and cruel has changed to insanity from the intolerable stings of a tortured conscience. It is sufficient to say, that this wretch was a ringleader in the mutiny of the Hermione frigate, in 1797, when the crew rose on the officers in the West Indies, and murdered them all except the surgeon and one master's mate. Notwithstanding all the precautions that have been taken, he has killed three persons since his confinement. How many he may have slain in honourable combat is unknown; but it is certain that after the horrible mutiny abovementioned, he fought along-side of Abercrombie in the battle of Aboukir, and Nelson in the battle of Trafalgar! The organ of *combativeness* ought to be strongly developed in this man.

One of the three murders alluded to, was committed in the present establishment. Having behaved well for some time, he was under no great restraint in April 1820, when he contrived to secrete the blade of an old knife, which he sharpened to a point, and watching an opportunity he plunged it twelve or fourteen times into the body of a poor sickly patient, named Dennis Leonard, with such force and fury that each stab would have killed a giant. After this he was hand-cuffed, but broke them into a thousand pieces. He is now confined in an iron cincture that surrounds his waist, with strong hand-cuffs attached to it.

"But his propensity to mischief, malice, and personal abuse, are as incessant as his taste for blood-shed and slaughter. He has contrived, notwithstanding his restriction of hands and feet, to break above seventy panes of glass, within the last two years, in the dining-room windows, although guarded on the inside by a strong iron wire lattice-work. This amusement he contrived to effect by standing on a form placed at some distance from the windows, and taking the bowl of his wooden spoon in his mouth, he poked the handle through the meshes of the wire-work, and thus broke the pane." 7.

Bloodshed and massacre are the constant topics of his frenzied discourse. His vengeance against poor Leonard was excited by some dispute about *Religion!* He told the coroner's jury, that if he could obtain the king's crown, and all the riches of the universe, he would not forego the pleasure of killing him, for all would be nothing to the ease of mind he felt in putting him out of the way!

"Even his very dreams, when he sleeps, are occupied with scenes of fury and vengeance; and he takes delight in detailing them the next morning. When he dreams of having murdered any, and sometimes all of the patients above named, he wakes quite pleased, and details the scene with much satisfaction; and the manner in which he has gratified his vengeance, and what fine fun he had in seeing them die. He thought he had a sword, with which he first cut all their throats, and then walked round them to see which should live longest; that when they were all dead, or nearly so, he split their skulls, and then transposed the brains from Hallwood's head into that of Lloyd; Lloyd's into Coates's; his to Jenkins's; his again into the Spaniard's, and his again to Hugh Dams's. He then ripped up their bellies, and changed their entrails in like manner; and then he hung and burned them all: but the only thing that grieved him was to hear them all talking in the gallery next morning. He stamps and raves most of the day, and nearly all night, with a piece of blanket crammed into his mouth, gnawing and tearing their souls out, as he imagines and terms it. He picks up pieces of glass, old nails, bones, and spoons which he grinds to a point, stones of a convenient size for flinging, and indeed every thing that is likely to enable him to do mischief, to which he is always inclined if he has an opportunity." 9.

There is a Greek confined in the same place, and nearly as furious as Walsh. The scenes that pass between these two, though at a distance, are terrific. The great wonder is, that Nature should be able to sustain such dreadful conflicts in the breast, with so little apparent effect on the corporeal fabric.

2. HATFIELD. This is the man who fired at His Majesty in Drury Lane Theatre, in the year 1800. It is curious that he was incited to this act by a fanatic who now inhabits the same asylum. Wounds which he had received in his head in the public service appear to have laid the foundation for his mental alienation, when he was persuaded to attempt the king's life to fulfil a prophesy of Bannister Truelock, a monomaniac to be noticed presently. This man, for a long time past, has evinced no symptoms of insanity; but his mind is soured, and he is discontented with every thing around him. Being a state prisoner, he will, of course, continue in his present abode for life.

3. TRUELOCK. This man, who was a shoemaker, having taken it into his head that the Messiah was to proceed from his mouth, in the same way that Johanna Southcote was to produce him from the uterus, persuaded Hatfield that the only obstacle was the king, who was first to be dispatched. He is a most singular character, and betrays no symptoms of insanity, unless the subject of RELIGION is touched upon, when adieu to all reason.

4. Mr. THOMAS LLOYD. This unparalelled compound of cunning, pride, wit, impudence, lies, filth, and phrenzy, was formerly a corn-factor in Mark Lane, and furnishes perhaps the most remarkable character that the annals of Bedlam are even doomed to record. A whole volume could scarcely contain a tenth of his eccentric operations from day to day. We cannot attempt to convey any idea of this non-descript lunatic to our readers. We shall only introduce a single extract as a sample.

"In the universality of his genius, he considers himself the most sublime poet that ever courted the muses, and whenever he can procure a scrap of paper, he proceeds to compose verses; but as those extemporaneous productions do not usually please his critic fancy, he converts them to ingredients in his gruel at breakfast, probably to enrich the soil of his genius for a new crop of heroics. This gruel is the common receptacle for a much greater variety of articles than usually go to the composition of turtle soup. He puts all his verses into it to cleanse them, as he says; and with a selection from the before-mentioned articles from his bosom, cheese from his snuff-box, butter from his tobacco-box, the exhausted quids from his mouth, sometimes the portion of another article that shall be nameless, bits of leather, small stones, pieces of bone, coals, &c. thus this *epicure* makes up for himself a relishing mess, somewhat a-kin to the hell-broth of Macbeth's wizards, and setting all German cookery at defiance. In this filthy system of culinary composition, however, he affects to proceed on scientific principles. Leather clarifies it; stones purify it; coals mineralize it; one article acidulates it; another gives it an alkaline virtue; a third, a high flavour. This mollifies it, that dulcifies it; the whole together makes it a delicious mess, and he swallows it with the *goût* of an Apicius. Sometimes his beer is enriched with part of the same ingredients; and though such is his daily practice, his health is not injured by it." 32.

5. LIEUT. TRAILE. We are next presented with a melancholy contrast to the facetious and satirical Lloyd. Mr. Traile served, during the late Peninsular war, as lieutenant in the 95th regiment of foot, with honour and reputation as a gentleman and an officer. At the peace he was placed on half-pay, and his mind soon afterwards became engloomed, partly from religious enthusiasm, and partly from imaginary apprehensions. He now became insane on a single point. He had a horror of death from poison in his food. He consequently came to the resolution of refusing all nutriment. Mr. Lawrence and Dr. Wright kept him alive for some time by forcing liquid food down his throat through a tube. On the 31st of March, 1822, he contrived to hang himself by means of his neck-handkerchief fastened to the wire-guard over the door. Thus he illustrated a very curious pathological fact in the history of insanity—namely, *suicide from fear of death*.

6. WILLIAM HOLWOOD. It is very astonishing what exertions and apparent—we might say real—sufferings will be borne by those affected with *nervous* diseases, and yet without the constitution experiencing any material injury. Instances of this kind must be familiar to every experienced practitioner—and they are constantly leading the younger branches of the profession into false prognosis and unnecessary alarm. The maniac abovementioned is an example. He is incessantly in action, night and day, "and yet it is astonishing how he has sustained so much exertion for so many years." He never seems exhausted—knows not what illness is—and appears at all times in robust health.

7. JOHN WILLIAMS. We only notice this case to express our approbation of some sensible observations of the anonymous author of the work before us, relative to the order of the Board at Bedlam, that forbids the sale, and consequently the use of tobacco among the insane.

"There can be no question that the forbidding this article is founded upon the best intentions; but there are many of the patients here who, from habits of ten, twenty, thirty, and even forty years' use of tobacco, in some shape or other, have contracted such an insuperable attachment to it, that they would much rather go without dinners than their beloved quid. Others prefer snuff, and those patients who have been long used to it are always discontented, fretful, and peevish, if debarred this comfort, either through the want of money to purchase, or the neglect of their friends to supply them with it. There seems to exist in the use of tobacco, as well as in madness, a pleasure which none but the infatuated know: and, trifling as the privation of those little enjoyments seems, it revives in the memory of the patients a sense of former grievances, gives them an idea that they are quite forsaken, renders them irritable, and has sometimes very injurious effects." 78.

8. GEORGE WOODS. This unfortunate gentleman was a surgeon, and consequently excites our sympathy in an unusual degree. His hallucination was, that he had undergone the fate of Abelard, and this loss was the subject of lamentation night and day. If any thing could excite our risible faculties, when such a melancholy subject as the loss of human reason is the topic, it is a scene which passed between poor Woods, his keeper, and a peeress who happened to be going round Bedlam in company with other ladies.*

This poor fellow, in allusion to the supposed loss of his testes, was constantly exclaiming "oh! my ——! what shall I do for my ——! they are gone! gone! for ever!

"The lady, unable to comprehend his meaning, applied for explanation to the keeper; who, with some discreet presence of mind told her ladyship that the poor patient was in the habit of collecting a quantity of pebbles, flints, and other rubbish about the airing ground, which he fancied to be emeralds, rubies, diamonds, and other precious gems; that it became necessary to take them away from him, in order to prevent the accumulation of such rubbish in the gallery; and that a recent privation of this sort was now the subject of his lament. 'Oh, poor man,' said her ladyship, turning to the patient, 'never mind them! When his lordship comes here again, he shall bring you some pretty ones which he has got at home; they will be much handsomer than those you have lost: so don't fret about them any more.' But poor Woods, not at all satisfied with the promised alternative, answered with much displeasure, 'that won't do! I will have none but my own.' And thus they separated, each unconscious of the other's meaning." 115.

9. JAMES W—L. We shall conclude our extracts with a passage from the case of this young gentleman, who was discharged cured in February 1822.

"The character and symptoms of this patient's disorder were extremely curious and singular. When the paroxysm came on, however he happened to be situated, his whole frame from head to foot became stiff, as if all his joints and muscles were ossified. His eyes, though staring open, became fixed, and he foamed at the mouth. If

---

* By the way, we think Bedlam a very unfit place for the visitations of our wives and daughters. They should be prohibited from beholding such scenes, and listening to such dialogues.

sitting or walking when his fit came on, he would instantly fall to the ground, generally extended at full length on his back, with the same symptoms of rigid stiffness and insensibility: his eyes, open and inclined upward, were insensible to the touch of a hand passed over them, which did not produce the slightest wink. No symptom of animation remained, with the exception of breathing; but this so faintly as to be scarcely perceptible. His condition in all other respects resembled death; and in this state he would sometimes continue for one, two, three, and even four days, without any apparent change. He could not be induced on those occasions to eat or take any kind of sustenance; except, under the direction of medical gentlemen, when rich broths were passed by injection sufficient to sustain nature: but of this he seemed quite unconscious. During the fits his whole person was literally as stiff as a plank, and he might be raised to a perpendicular, and carried from place to place like a ladder, without the least appearance of flexibility. Towards the termination of those paroxysms, when a hand was passed over the eye-balls they would sometimes move, which was a prognostic of his recovery: and on being roused from his stupor, he recollected nothing of what had passed; but he would speak of dreams, visions, heaven, hell, and the strange things he had seen, at least with 'his mind's eye.' Visions which, with the fanatical disciples of Hannah Southcote, would have stamped him a sealed prophet, and sanctioned as divine revelations the wild dreams of his disordered fancy. After those fits he always appeared weak and dejected." 157.

Here we must conclude our notice of this work, having given our readers sufficient specimens of its nature and execution. We have one or two remarks to make in parting with the anonymous author. We much question the propriety or the right of thus publishing the name, histories, and unfortunate conditions of the inhabitants of this asylum for the wreck of human intellect. We have been told that the author is not attached to the establishment; but we are convinced that no mere visitor could have collected such a mass of authentic names, dates, and minute circumstances, without *assistance* from some of the residents. The publication, then, we deem a breach of trust of a very reprehensible nature, not only as exposing to public curiosity what ought not to be blazoned to the world—but as tending to injure, or at least curtail the utility of the excellent establishment which the author has so much and so justly praised. Few people will like to send their friends to a place where their histories may probably be taken down and committed to the press. Neither can we commend the tone of levity, and the sallies of wit or humour which pervade these "Sketches in Bedlam." The loss of reason is no joke; and although the poor lunatic may laugh at his own degraded condition, we deem it bad taste in the bye-stander to join in the jest.

